# High Selectivity and Yield in Catalytic Transfer Hydrogenation of Furfural to Furfuryl Alcohol by Zirconium Propoxide Modified Mesoporous Silica

**DOI:** 10.3390/molecules30173600

**Published:** 2025-09-03

**Authors:** Agnieszka Ciemięga, Katarzyna Maresz, Katarzyna Janoszka, Julita Mrowiec-Białoń

**Affiliations:** 1 Institute of Chemical Engineering of the Polish Academy of Sciences, Bałtycka 5, 44-100 Gliwice, Poland; maresz@iich.gliwice.pl (K.M.); j.bialon@iich.gliwice.pl (J.M.-B.); 2 Institute of Environmental Engineering of the Polish Academy of Sciences, M. Skłodowskiej-Curie 34, 41-819 Zabrze, Poland; katarzyna.janoszka@ipispan.edu.pl

**Keywords:** furfural transformation, Lewis acid sites, MPV reduction

## Abstract

The aim of the work was to develop a highly effective catalyst for the conversion of furfural into furfuryl alcohol through catalytic transfer hydrogenation, which is an important process for converting biomass-derived compounds into valuable chemicals. A highly mesoporous silica was modified with various zirconium and aluminium precursors to obtain Lewis acid centres. The materials were characterised by nitrogen adsorption, FTIR spectroscopy, pyridine adsorption, thermogravimetry, SEM and XRD. The catalytic properties of the materials versus acid site concentration, alcohol type, zirconium content and reaction time were investigated in a batch reactor. The zirconium propoxide-modified materials appeared to be the most active and selective catalysts in the reaction studied. They showed complete furfural conversion with ca. 99% selectivity of furfuryl alcohol, which was attributed to the predominantly Lewis acidic character of these catalysts. High productivity, 15.2 mol_FA_/mol_Zr_·h, was obtained for the most active catalyst. Good catalytic stability was confirmed in repeated cycles. The oxide form of zirconium and aluminium species resulted in the mixed Lewis and Brönsted acidity, which encouraged further transformation of furfuryl alcohol into butyl furfuryl ether, angelica lactone and butyl levulinate. The elaborated catalyst offers a promising approach for converting renewable resources into industrially relevant chemicals.

## 1. Introduction

The increasing demand for sustainable chemical processes and the depletion of fossil resources have intensified the search for renewable carbon-based alternatives. Lignocellulosic biomass, due to its abundance and renewability, has become a key feedstock in the production of biobased platform chemicals such as cyclopentanone, γ-valerolactone, furans [1,2,3]. Among these, furfural has attracted significant attention as a versatile intermediate for the synthesis of a wide range of chemicals. Furfural is produced on an industrial scale mainly from the hemicellulose fraction of biomass through the acid-catalysed dehydration of xylose [4,5,6]. One of the most important subsequent transformations of furfural is its hydrogenation to furfuryl alcohol. This alcohol is used as a precursor for various industrial applications, including resins, polymers, adhesives, lubricants, and drug intermediates such as vitamin C, lysine and ranitidine [7,8,9,10,11,12,13]. Traditionally, the hydrogenation of furfural is carried out using copper–chromite catalysts under high-pressure hydrogen and elevated temperatures. Despite their industrial relevance, the use of hexavalent chromium causes serious environmental and health concerns due to its toxicity. Furthermore, the need for external molecular hydrogen introduces additional safety, economic, and logistical issues [14]. Catalysts based on noble metals such as palladium, platinum, ruthenium, and rhodium have been studied extensively and show high hydrogenation activity [15,16,17]. However, their practical implementation is limited by high cost, scarcity and high sensitivity to poisoning. Moreover, the use of these catalysts can lead to the formation of undesired reaction products, such as tetrahydrofurfuryl alcohol or 2-methylfuran. Nickel-based systems demonstrate good hydrogenation performance, but often require elevated temperature, which can result in deactivation through sintering. Moreover, the process requires hydrogen and high pressure [18,19]. To overcome some of these limitations, research has shifted toward the development of alternative catalytic systems capable of operating under milder and more environmentally friendly conditions.

A promising strategy for the reduction of furfural to furfuryl alcohol is catalytic transfer hydrogenation via the Meerwein–Ponndorf–Verley (MPV) reduction. In this reaction, a secondary alcohol is used as both the solvent and hydrogen donor, transferring a hydride to the carbonyl group of furfural in the presence of a Lewis acid catalyst [20]. The efficiency and selectivity of the MPV reaction strongly depend on the Lewis acid catalyst employed. Zirconium-based catalysts exhibit strong Lewis acidity and high thermal stability, which facilitate the activation of the carbonyl group in furfural [21]. Materials such as Zr-Beta zeolites and Zr-doped silicas have shown high catalytic activity, especially when the zirconium is well dispersed in the framework [22,23,24,25,26]. Alumina-based catalysts, including amorphous Al_2_O_3_ and aluminosilicates, offer moderate Lewis acidity and are easily available, but typically show lower activity and selectivity than zirconium analogues [21,27]. Bifunctional catalysts that incorporate two or more metal oxides, such as Mg-Fe, Zr-Ti, or Mg-Ti systems, have also been explored to combine the advantages of each component and improve catalytic performance. While such combinations can exhibit synergistic effects, their synthesis is often more complex [28]. More attention has been paid to materials with tailored porosity and high surface area. Mesoporous silicas, such as SBA-15 or MCM-41 functionalized with Lewis acidic metal centres, provide improved mass transfer and site accessibility [29,30]. Additionally, metal–organic frameworks (MOFs) such as UiO-66 and MIL-140, have shown promise as MPV catalysts due to their tuneable pore structures and well-defined active sites [31,32].

The selection of the hydrogen donor is also critical to the performance of MPV-type hydrogenation. Secondary alcohols such as isopropanol and 2-butanol are typically used because of their high hydrogen transfer efficiency [33].

One of the key challenges in the reduction of furfural to furfuryl alcohol using MPV reaction is product selectivity control. Furfural can undergo subsequent transformations, such as etherification and esterification, which lead to the formation of alkylfurfuryl ethers or esters, especially in the presence of strong protic acid sites [34,35,36]. Although these compounds may be valuable in certain applications, their formation complicates the purification process and reduces the overall efficiency, particularly when furfuryl alcohol is the target product. Therefore, the search for highly selective catalysts that limit secondary transformations is of considerable interest.

In this study, we report the synthesis and application of a ZrOPr-doped silica catalyst that features well-dispersed and low content Zr^4+^ Lewis acid sites grafted onto the mesoporous silica support. This catalyst enables the efficient and selective MPV reduction of furfural to furfuryl alcohol under mild conditions using 2-butanol as the hydrogen donor. The performance of the catalyst was compared with those of zirconia and alumina modified support and with data published in the literature. Moreover, the effects of zirconium concentration, alcohol type, and reaction time on furfural chemical transformation pathways were investigated. The kinetics of MPV reduction of the most effective catalysts was considered.

## 2. Results

### 2.1. Properties of the Materials

All synthesised materials were used in the form of powders with grains of size in the range of 20 to 50 μm. An irregular shape is well seen in the SEM image of 7ZrOPr-S sample (Figure S1). XRD analysis showed amorphous structure of the catalysts. The spectrum of a representative sample was presented in Figure S2, and it shows only broad peak in the range of 15 to 30 2Theta, which is characteristic for amorphous silica.

**Figure 1:**
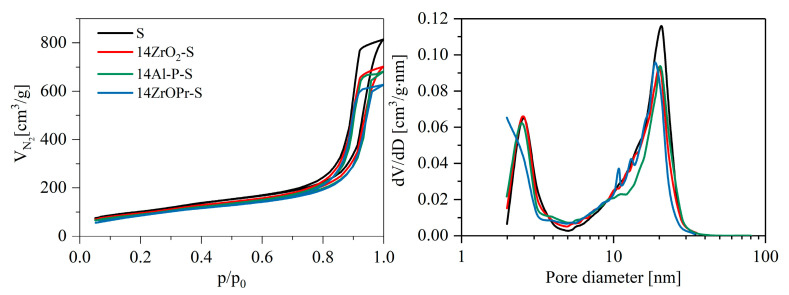
Nitrogen adsorption/desorption isotherm of pristine and modified silica.

Nitrogen adsorption–desorption isotherms (Figure 1) were collected to investigate the impact of surface modification on the structural properties of mesoporous silica. The specific surface area S_BET_ and the pore volume V_p_ of the silica support before and after its functionalisation with active species were determined and summarised in Table 1 for representative samples.

The pristine silica showed a typical type IV isotherm with an H1 hysteresis loop according to the IUPAC classification, characteristic of mesoporous material with cylindrical pores and a uniform pore size distribution. It exhibited a high specific surface area of 358 m^2^/g and a total pore volume of 1.23 cm^3^/g, with mesopores of 2.5 and 20 nm in diameter. The incorporation of aluminium and zirconium species led to a slight decrease in surface area of about 6% in all samples. After modification, the bimodal pore size distribution was preserved. However, the distribution of the smaller mesopores shifted slightly toward smaller diameters. The total pore volume decreased by c.a. 15% in oxide-modified materials, and by 23% in those modified with zirconium propoxide.

**Figure 2:**
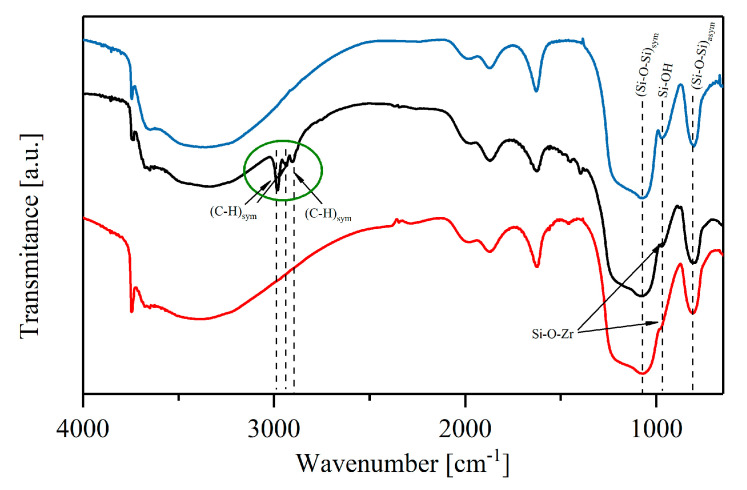
FTIR spectra of silica (blue), 14ZrOPr-S (black) and 14ZrO_2_-S (red) samples.

Figure 2 presents the FTIR spectra of 14ZrOPr-S and 14ZrO_2_-S samples. The spectrum of the zirconium propoxide-modified material (black) shows distinct bands at 2980 and 2940 cm^−1^, as well as 2900 cm^−1^, corresponding to the asymmetric and symmetric C-H stretching vibrations of the isopropyl groups. It indicates the presence of the ligands coordinated to zirconium centres. These C-H bands are not observed in the zirconia-modified silica spectrum (red) confirming the complete thermal decomposition of these groups. The spectra of both catalysts feature a broad band centred at 3400 cm^−1^, attributed to hydrogen-bonded hydroxyl groups. The bands observed in the range of 3600–3800 cm^−1^ were ascribed to isolated Si-OH and Zr-OH. These bands are more pronounced and sharper for the red spectrum, indicating a higher density of Brönsted acidic centres. In the mid-IR region 1300–1000 cm^−1^, both spectra exhibit intense bands characteristic of Si-O-Si asymmetric 1100 cm^−1^ and symmetric 800 cm^−1^ stretching vibrations. The absorption band corresponding to stretching modes of the Si-O-Zr bridges can be seen at 960 nm^−1^ [37,38,39,40].

**Figure 3:**
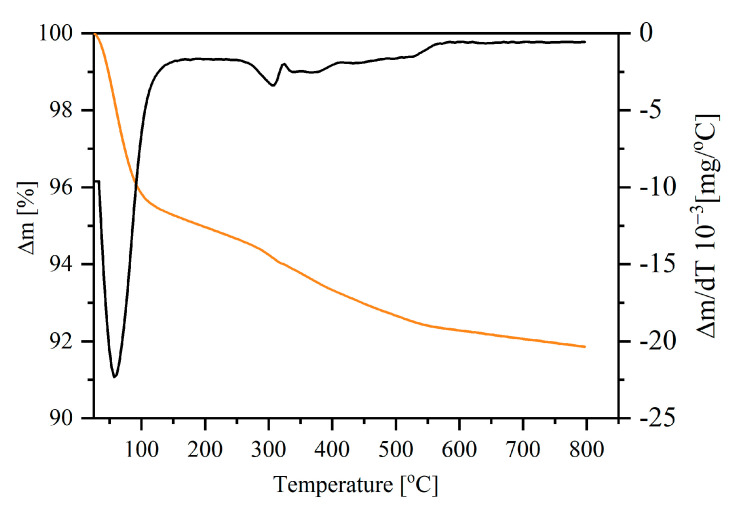
TG (black) and DTG (orange) curves of 14ZrOPr-S.

Thermogravimetric analysis was performed to determine the thermal stability and amount of the propoxy groups in the functionalised materials. Figure 3 shows the weight loss vs. temperature curve for the 14ZrOPr-S sample. The TGA curve revealed two regions of mass loss. The initial weight loss of approximately 5% occurred in the range of 25 to 150 °C and is attributed to the removal of physisorbed water from the surface. The second, corresponding to the 3% decrease, was observed between 250 and 600 °C. This loss is associated with the gradual thermal decomposition of organic ligands bonded to zirconium ions. The amount of propoxy ligands was estimated and the average number of -C_3_H_7_ per zirconium atom was calculated to be approximately 1.13. This suggests that a part of the ligands was hydrolysed to form -OH groups.

**Figure 4:**
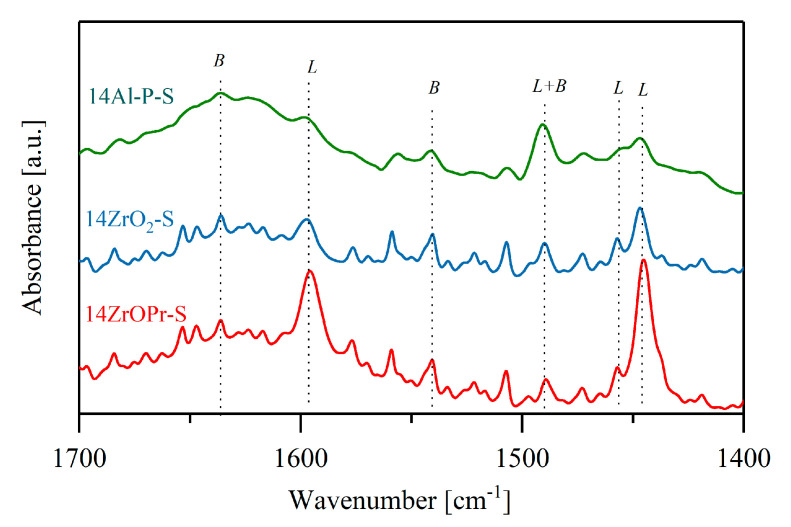
FTIR spectra of 14ZrOPr-S, 14ZrO_2_-S and 14Al-P-S catalysts after pyridine adsorption.

FTIR spectra (Figure 4) of pyridine adsorbed on silica-based materials modified with 14ZrOPr-S, 14ZrO_2_-S, and 14Al-P-S provide information on the type of acid sites. Characteristic bands at 1540 cm^−1^ and 1635 cm^−1^ are attributed to pyridine coordinated to the Brönsted (B) acid sites, while bands at 1445 cm^−1^, 1456 cm^−1^, 1600 cm^−1^ are observed for pyridine adsorbed on Lewis acid sites. An additional band at around 1490 cm^−1^ is associated with contributions from both Brönsted and Lewis sites (L + B) [41,42].

Spectra of all analysed samples exhibit bands characteristic of both Lewis and Brönsted acid sites; however, their relative intensities differ significantly. The ZrOPr-modified silica shows well-defined bands attributable to Lewis acid sites. This indicates that zirconium is anchored to the silica matrix through the Si-O-Zr bond as an unsaturated coordinating centre that acts as an electron pair acceptor. The relatively low Brönsted acidity in this sample suggests that this material can be considered as a selective Lewis acid catalyst.

In the 14ZrO_2_-Si spectrum, the presence of a strong band near 1540 cm^−1^ and 1603 cm^−1^ implies that the surface hydroxyl groups associated with ZrO_2_ domains act as proton donors capable of forming pyridinium species. The catalyst still possesses a strong and dominant Lewis acidic character, although it is weaker than that of the 14ZrOPr-Si material.

The Al-modified silica exhibits the most intense Brönsted acid band, indicating a high concentration of protonic sites. A Lewis acid signal is also observed, which confirm the presence of unsaturated coordinating aluminium species.

The relative concentration ratio of the L/B sites was determined and collected in Table 2. This ratio was calculated from the integration of the bands at 1445 cm^−1^ and 1540 cm^−1^ assuming the extinction coefficient ε = 2.22 cm/μm and ε = 1.67 cm/μm, respectively [43]. These results show that Lewis acidity decreased in order 14ZrOPr-Si > 14ZrO_2_ > 14Al-P-S, and in the alumina-functionalised sample the highest concentration of Brönsted acid sites was observed.

### 2.2. Catalytic Performance of the Materials in the MPV Reduction

**Scheme 1:**
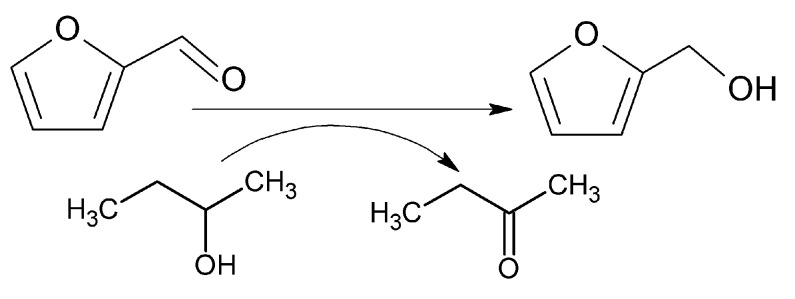
Chemoselective MPV reduction of furfural to furfuryl alcohol with 2-ButOH.

The catalytic performance of the catalysts was investigated in the Meerwein–Ponndorf–Verley (MPV) reduction of furfural (FF) using 2-butanol as the hydrogen donor. The reaction scheme is depicted on Scheme 1.

**Scheme 2:**
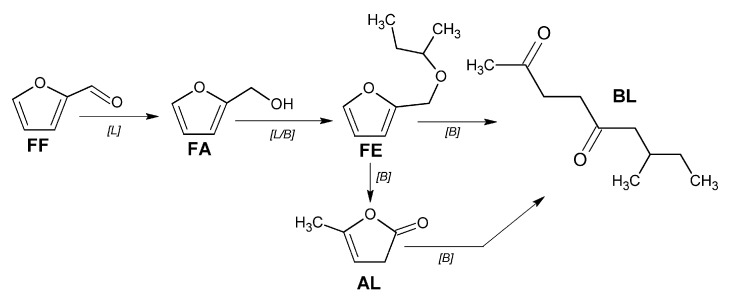
Reaction pathways of acid-catalysed conversion of furfural, Lewis [L], Brönsted [B], mixed Lewis and Brönsted [L/B] acid sites.

The target product of this reaction is furfuryl alcohol (FA), which can, however, undergo further acid-catalysed transformations into butyl furfuryl ether (FE), butyl levulinate (BL) and angelica lactone (AL) as shown in Scheme 2. The sequential reaction pathway follows the transformation of FF to FA via Lewis acid-catalysed hydride transfer, followed by Brönsted acid-catalysed etherification of FA to FE. Subsequently, FE can be converted to AL or BL, while AL may further react with 2-butanol to form BL.

**Figure 5:**
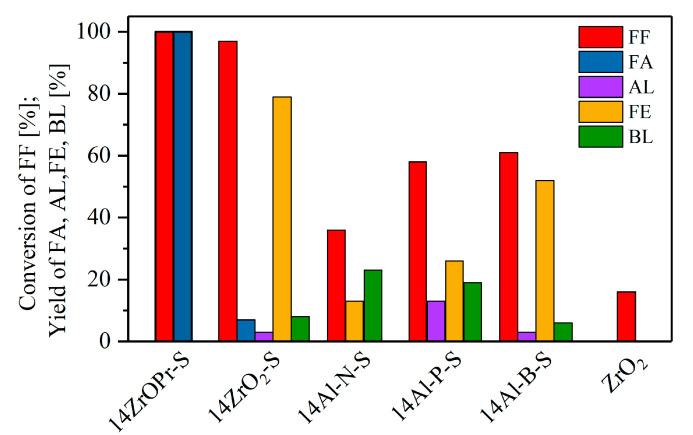
Conversion of furfural and yield of reaction products obtained for Zr- and Al-catalysts.

Figure 5 shows conversion and selectivity in catalytic transformation of furfural by mesoporous silica support modified with zirconium and aluminium. The 14ZrOPr-Si catalyst demonstrated 100% conversion of FF and 100% yield of FA. The MPV reduction mechanism proceeds through a six-membered transition state in which the furfural carbonyl group coordinates with a Lewis-acidic Zr^4+^ centre, facilitating hydride transfer from the secondary alcohol. The course of the reaction is in agreement with the predominance of Lewis acid sites in this material, which was proved by FTIR spectroscopy (see analysis of pyridine adsorption). The 14ZrO_2_-modified silica also showed almost 100% furfural conversion. However, only a 10% yield of FA was detected, and the yield of the main product FE was 80%. In the post-reaction mixture minor amounts of ester BL and lactone AL were also identified. The presence of these products was attributed to the coexistence of both Lewis and Brönsted acid sites.

The alumina-modified silica catalysts exhibited a stronger Brönsted acid character and a lower activity in the MPV step. The catalyst obtained from aluminium nitrate showed only 38% FF conversion and FA was not observed because of its rapid conversion into secondary products. A similar course of chemical transformations was observed for the catalysts 14Al-P-S and 14Al-B-S prepared from aluminium alkoxides, but higher conversion of FF was achieved, at 55% and 57%, respectively. The reaction products were FE, Al and BL with different yields, suggesting the presence of Brönsted acid centres of different concentrations/strengths in these materials. The commercial ZrO_2_ catalyst demonstrated very low conversion of furfural, about 15%, and FA, FE, AL, or BL were not detected. In the presence of bulk zirconia, furfural may be initially reduced to FA, which is rapidly consumed through undesired side reactions, i.e., polymerisation into oligomeric or polymeric species. Alternatively, furfural may also undergo condensation reactions, leading to the formation of humin-type solids [44].

The ability of the catalyst to promote the MPV reduction of furfural strongly depends on the coordination of the metal centre anchored to the silica surface. In Zr-propoxy-derived materials, Zr centres exist as isolated, tetra-coordinated Lewis acidic sites and this geometry enables the formation of a six-membered cyclic transition state [40]. In zirconia-deposited silica, Zr species may form oligomeric Zr-O-Zr domains, which restrict the creation of Lewis acid centres. The use of aluminium alkoxide precursors results in weak Lewis acidity, while the application of aluminium nitrate can lead to the formation of alumina clusters and the formation of more Brönsted acid centres than desired Lewis ones [45,46].

**Figure 6:**
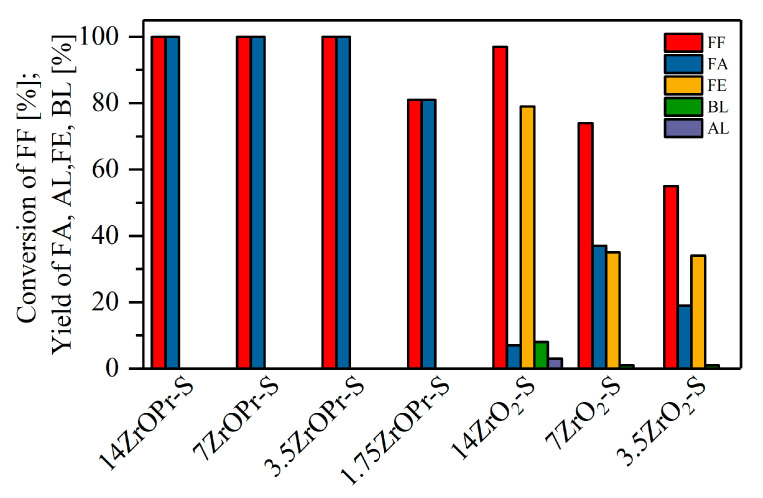
Influence of Zr concentration on FF conversion and selectivity.

The effect of zirconium concentration on the efficiency of MPV reduction was studied for two most active and selective systems: silica-supported zirconium propoxy species with dominant Lewis acidity and silica-supported zirconia containing both Lewis and Brönsted acid sites (Figure 6). ZrOPr-S catalysts showed high activity and selectivity for MPV reduction of furfural to furfuryl alcohol, regardless of metal loading. Full conversion of FF and ca. 99% yield of FA was reached after 6 h for 14Zr-, 7Zr- and 3.5Zr-catalysts. At the lowest loading of zirconium, high FA selectivity was retained; however, FF conversion declined to 81%. A strong decrease in catalytic activity versus lowering Zr content was observed for zirconia-based catalysts. Moreover, low selectivity towards FA was detected for these materials.

**Figure 7:**
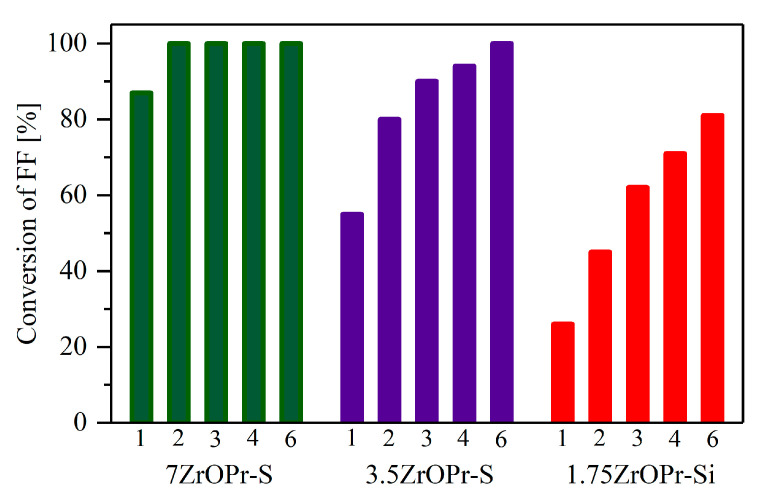
Conversion of FF and selectivity to FA vs. time for ZrOPr-Si catalysts. Selectivity of FA in all experiments was ca. 99%.

The progress of MPV reduction versus time was investigated for three catalysts of the ZrOPr-S series and presented in Figure 7. It shows that almost full conversion of FF was already obtained after 2 h for the 7ZrOPr-S catalyst. These data clearly indicated the high activity and selectivity of zirconium-propoxide supported on the mesoporous silica at its relatively low concentration.

**Figure 8:**
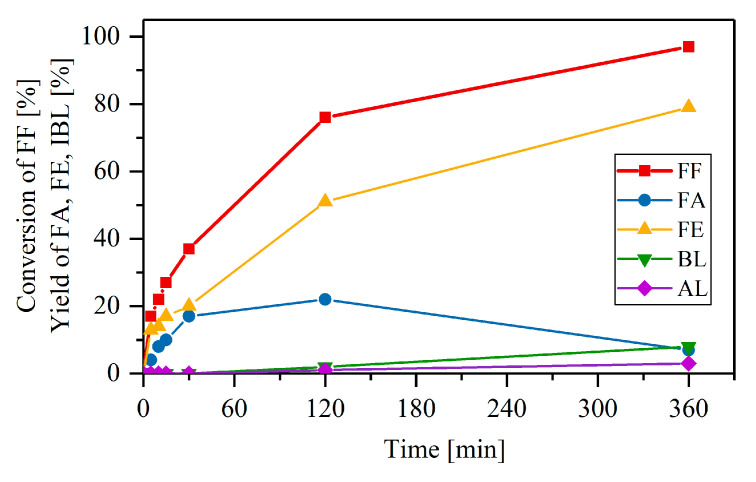
Conversion of FF and yield of FA, FE, Al, IBL vs. time for 14ZrO_2_-S catalyst.

The presence of a fairly high concentration of Brönsted acid sites (see Table 2) in zirconia-functionalised materials has strong impact on the chemical transformation of FF as was shown in Figure 5 in a process carried out for 6 h. Therefore, the changes in substrate and products concentration over time were investigated (Figure 8). The FA product of the MPV reduction was rapidly converted to FE, and its concentration was proportional to the FF consumption. As the chemical reaction progressed, a small fraction of FE was reacted to BL with 5% yield after 6 h. The predominance of FE among the products indicated that the Brönsted acidity in this catalyst is sufficient to drive FA conversion (see Scheme 2) but not enough to complete the transformation to AL or BL under the reaction conditions used. A high FE yield of about 80% was achieved after 6 h, highlighting the potential of ZrO_2_-S catalyst in synthesis of this valuable chemical compound [47].

The hydrogen donor has an evidenced effect on the conversion of furfural during MPV reduction. Table 3 shows the results of catalytic tests for the 3.5ZrOPr-S catalyst after 1 h of reaction carried out at 120 °C. 2-Butanol provided the highest conversion, followed by 2-pentanol and 2-propanol. These results are consistent with previous reports [48,49] and the generally accepted MPV reduction mechanism, in which the hydride is transferred from the α-carbon of the alcohol to the carbonyl carbon of the aldehyde and the resulting complex stabilises more efficiently with secondary alcohols. In addition, they exhibit more favourable thermodynamics and kinetics for hydride donation, since they form more stable ketones compared to aldehydes derived from primary alcohols (2-propanol is oxidised to acetone, 1-propanol to propionaldehyde) [50]. In contrast, 2-heptanol resulted in a dramatically reduced conversion likely due to its insufficient polarity, and also to the shielding effect of the active centre [51]. It is important to note that differences in catalytic activity cannot be ascribed to steric hindrance within the pores of the catalyst. The catalyst possesses sufficiently large pore sizes to accommodate bulky molecules and to allow the formation of a transition state.

**Figure 9:**
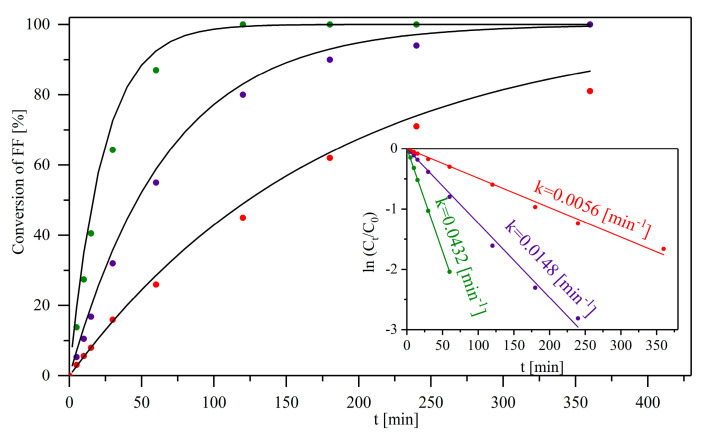
Conversion of furfural MPV reduction vs. time and ln (C_0_/C) vs. time plot (inset) for 7ZrOPr-S (green), 3.5ZrOPr-S (purple), and 1.75ZrOPr-S (red) catalysts. Points represent experimental data and lines model.

The kinetics of MPV reduction of furfural was approximated by a pseudo-first-order kinetic model with respect to furfural concentration, because of a large excess of reducing alcohol was used in the reaction. The experimental data fit the kinetic model very well (R^2^ = 0.995) (Figure 9). The apparent rate constants for 7ZrOPr-S, 3.5ZrOPr-S and 1.75ZrOPr-S catalysts were determined and the values of 0.0432, 0.0148, and 0.0056 min^−1^ were obtained, respectively. The rate constant of the 7ZrOPr-S catalyst is comparable to the values reported in the literature, which range from 0.01 to 0.04 min^−1^ [24,30,52,53].

**Figure 10:**
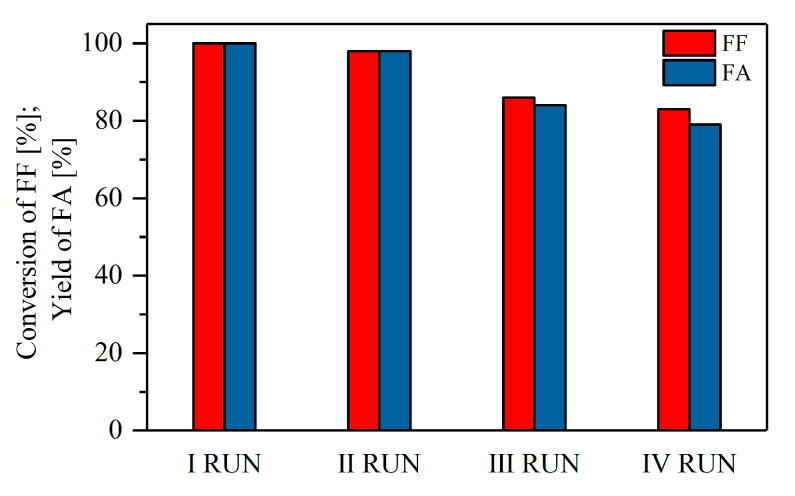
Catalyst recyclability test: 7ZrOPr-S catalyst, 2-buthanol, reaction time 2 h.

The stability of the 7ZrOPr-Si catalysts was checked in four cycles (Figure 10), and it showed a 13% decrease in furfural conversion after the third cycle, as well as an additional decrease, ca. 4%, after the fourth cycle. A slight reduction in FA yield was also observed. This was a consequence of the blocking of active sites by adsorbed molecules. The thermogravimetric analysis (Figure S3) revealed an increased mass loss of the spent catalysts. The most pronounced adsorption of molecules was observed after the first run (approximately 1.6%). Similar observations have been reported by other researchers, who associated the decrease in catalytic activity with the accumulation of humins [31,54,55,56].

The catalytic performance of our best catalyst 7ZrOPr-Si has been compared in terms of furfural conversion, furfuryl alcohol selectivity, and productivity, with various zirconium-based catalysts presented in the literature (Table 4). It demonstrated excellent catalytic performance under relatively mild conditions using 2-butanol as the hydrogen donor. The concentration of by-products was below the limit of quantification.

## 3. Materials and Methods

### 3.1. Materials

Polyethylene glycol 35,000, (Sigma-Aldrich, St. Louis, MO, USA), tetraethoxysilane (TEOS, 99%, ABCR, Karlsruhe, Germany), HNO^3^ (65%, Avantor, Radnor, PA, USA), cetyltrimethylammonium bromide (CTAB, Sigma-Aldrich, St. Louis, MO, USA), ammonia solution (25%, Avantor, Radnor, PA, USA), zirconium propoxide (70% in 1-propanol), 1-propanol (Avantor, Radnor, PA, USA), aluminium nitrate nonahydrate (Avantor, Radnor, PA, USA), aluminium di (isopropoxide) acetoacetic ester chelate (>98%, Aldrich, Milwaukee, WI, USA), aluminium di-sec-butoxide ethyl acetoacetate (95%, ABCR, Karlsruhe, Germany), zirconia (Aldrich, Milwaukee, WI, USA), 2-butanol (Avantor, Radnor, PA, USA), 1-butanol (Roth), 2-pentanol (ThermoScientific, Waltham, MA, USA), 2-propanol (ChemSolute, Renningen, Germany), 2-heptanol (Fluka, Buchs, Switzerland), furfural (99%, Sigma-Aldrich, St. Louis, MO, USA), furfuryl alcohol (98%, Acros, Ghent, Belgium), and dodecane (Avantor, Radnor, PA, USA) were used as received.

### 3.2. Synthesis of Catalysts

Mesoporous silica materials were synthesised using a soft-templating sol–gel approach inspired by the method originally reported by Nakanishi and subsequently modified in several studies [60,61,62]. In a typical synthesis, polyethylene glycol (PEG, M_n_ = 35,000) was dissolved in 1 M nitric acid under continuous stirring to ensure complete homogenisation. The resulting solution was cooled in an ice bath and tetraethyl orthosilicate (TEOS) was added dropwise as the silica source. The mixture was stirred for one hour at room temperature to initiate hydrolysis and condensation reactions. Subsequently, cetyltrimethylammonium bromide (CTAB) was introduced as the mesostructure-directing agent to promote the development of mesoporosity. The sol was then transferred into polypropylene moulds and statically aged at 40 °C for 8 days. After removal from the moulds, the wet silica was subjected to a hydrothermal treatment in 1 M aqueous ammonia at 90 °C for 9 h. The material was then washed with distilled water, air-dried at 40 °C, and calcined in air at 550 °C for 5 h to remove organic templates.

The post-synthesis functionalisation of the silica with zirconium species was carried out through a grafting procedure using zirconium (IV) propoxide. Prior to modification, the silica supports were dried at 200 °C under dry nitrogen to eliminate physiosorbed moisture and prevent uncontrolled hydrolysis of the zirconium alkoxide. The grafting was carried out by soaking the silica material in a solution of zirconium precursor in 1-propanol. Impregnation was performed at 70 °C for 24 h under static conditions. The material was dried at 110 °C in a nitrogen flow and ground into fine powder.

The zirconia–silica material was obtained by calcining the zirconium–propoxide-doped silica at 550 °C in air. Al_2_O_2_-SiO_2_ samples were prepared by impregnation of silica mesoporous material with solution of aluminium alkoxides or aluminium nitrate in parent alcohol or water, respectively, using a procedure similar to that for zirconium modified materials. Finally, they were calcined at 500 °C. The samples were denoted as xZry-S and xAl-z-S, where x is the mass (g) of Zr or Al per 100 g of Si, the y-ligand (OPr or O_2_), and z signifies the aluminium isopropoxide ethyl acetoacetate, aluminium di-sec-butoxide ethyl acetoacetate, or aluminium nitrate, indicated by the abbreviation P, B, and N, respectively. S stands for silica support.

### 3.3. Characterisation Techniques

The textural properties of the synthesised materials were determined from low temperature nitrogen adsorption/desorption isotherms obtained at −196 °C using a ASAP 2020 apparatus (Micromeritics, Norcross, GA, USA). Prior to analysis, the samples were outgassed under vacuum at 200 °C for 24 h. The specific surface area (S_BET_) was calculated using the Brunauer–Emmett–Teller (BET) method, while the mesopore volume and pore size distribution were determined from the desorption branch of the isotherm using the Barrett–Joyner–Halenda (BJH) model. Thermal analysis of the zirconium-modified samples was performed on a STAR 850 instrument (Mettler Toledo, Greifensee, Switzerland) to determine the thermal stability and the content of organic ligands. The measurements were carried out in air with a flow rate of 60 cm^3^ min^−1^ in the range of 25–800 °C, applying a heating rate of 10 K min^−1^. The presence of propoxy moieties was additionally confirmed by FTIR spectroscopy. The spectra were collected using the DRIFT technique. The types of acid sites on the surface of modified silica were studied by FTIR spectroscopy (Nicolet 6700, Thermo Scientific, Madison, WI, USA) using pyridine as a probe molecule. Before pyridine adsorption, the samples were dried at 110 °C. Pyridine vapour was introduced at room temperature and allowed to equilibrate for 2 h. Subsequently, physically adsorbed pyridine was removed by evacuation at 150 °C for 30 min. The spectra were recorded using a high-temperature vacuum cell equipped with ZnSe windows. The scanning electron microscopy images were obtained in PhenomPure instrument (BSD detector, voltage 5 kV, gold layer 3 nm, Thermo Scientific, Waltham, MA, USA). The X-ray diffraction pattern of the sample was collected by a PAN analytical X’Pert Pro PW3040/60 diffractometer for 2theta in the range of 10–60 with increment of 0.03°/step (Malvern, UK). The ICP analysis was used to determine the concentration of Zr and the details can be found in Ref. [40].

### 3.4. Catalytic Activity Experiments

In a typical reaction test, a mixture containing 0.22 mmol of furfural, 22 mmol of 2-butanol, dodecan as an internal standard, and 0.0279 g of catalysts was placed in a tightly sealed vial and heated at 120 °C. The temperature was thermostatically controlled by an oil bath, and the mixture was continuously stirred at 500 rpm. After desired time, the vial was immediately cooled, the catalyst was separated and the reaction solution was analysed by chromatography (Agilent 7980A, HP-5 capillary column, FID detector, Agilent Technologies, Inc., Santa Clara, CA, USA). The qualitative analysis of the reaction products was carried out using GC-MS (Thermo Scienific Trace 1300 coupled with an ISQ7000 mass spectrometry detector, TG-5MS capillary column, Thermo Scientific, Waltham, MA, USA). The standard deviation in catalytic experiments was less than 3%, and the bars were not included in the graphs since they were hardly seen.

## 4. Conclusions

The performance of zirconium and aluminium-modified silica mesoporous materials in the conversion of furfural to furfuryl alcohol through catalytic transfer hydrogenation strongly depended on the ratio of the Lewis to Brönsted acid sites. It has been shown that mesoporous silica grafted with zirconium propoxide had a higher concentration of Lewis acid centres than those of zirconia and alumina modified materials, which resulted in the conversion of furfural to the desired alcohol with almost 100% selectivity. The conversion depended on the zirconium concentration, the type of alcohol used as a hydrogen donor, and the duration of the catalytic process. The pseudo-first-order kinetic equation describes the course of the reaction with high accuracy, and the value of apparent rate constant confirmed the high performance of the best catalyst, i.e., 7ZrOPr-S sample. The presence of a relatively large number of Brönsted acid centres in zirconia and alumina-modified catalyst led to further chemical transformation of furfuryl alcohol. The butyl furfuryl ether, angelica lactone, and butyl levulinate were obtained with different yield depending on the zirconia content and type of alumina precursor used in the catalyst’s synthesis. A decrease in furfural conversion was observed after the third cycle, and a slight reduction in yield was also recorded. Moreover, the highly porous structure of the catalyst, with mesopores of 20 nm in diameter, does not restrict the transport of large molecules to the active centres and allows the formation of the transition state of MPV reaction.

## Figures and Tables

**Table 1:** Structural parameters of materials.

Support/Catalyst	S_BET_ [m^2^/g]	V_p_ [cm^3^/g]
S	358	1.23
14ZrOPr-S	337	0.95
14ZrO_2_-S	340	1.06
14Al-P-S	330	1.04

**Table 2:** Relative ratio of Lewis to Brönsted acid sites.

Catalyst	Relative L/B Ratio
14ZrOPr-S	8.5
14ZrO_2_-S	2.5
14Al-P-S	1.3

**Table 3:** MPV reduction of furfural with the use of primary and secondary alcohols.

Solvent	b. p. [°C]	ε	Conversion of FF [%]
2-Propanol	82	18.3	36
2-Butanol	99	15.8	55
2-Pentanol	119	13.6	38
2-Heptanol	159	9.21	3
1-Propanol	97	20.1	6
1-Butanol	118	17.8	10

**Table 4:** Conversion of furfural and productivity/selectivity of furfuryl alcohol for zirconium-based catalyst.

Catalyst	Temp. [°C]	Time [h]	Solvent	FF Conv. [%]	P *	FA Selec. [%]	Ref.
Zr@OMCs	180	4	2-PrOH	100	3.75	93.2	[54]
Zr(OH)4	170	2.5	2-PrOH	100	0.01	99	[55]
Zr-LS	100	1	2-PrOH	97.5	4.37	98.5	[57]
Zr5-GAF	100	1	2-PrOH	96.5	13.2	98.1	[58]
meso-Zr-glu	180	3	2-PrOH	100	2.52	92	[56]
M-MOF-808	100	2	2-PrOH	99.2	17.6	93.8	[31]
UiO-66	150	6	2-PrOH	82.1	N/A	~92	[32]
ZrO_2_-SBA-15(1)	130	1.5	2-PrOH	75	9.28	45	[53]
Zr1-SBA-15	120	3	2-PrOH	99	10.38 **	98.5	[30]
Zr-SBA-15_25	190	1	2-PrOH	>99	N/A	N/A	[59]
RT (Zr-SBA-15)	110	4	2-BuOH	95	3.85	74	[23]
ZrO_2_(20)@SBA-15	140	3	2-PrOH	93.2	10	96.9	[24]
Zr-MCM-41	130	24	2-PrOH	96.9	N/A	N/A	[29]
7ZrOPr-Si	120	1.5	2-BuOH	99	15.2	>99	This work

* Productivity: mol_FA_/mol_Zr_·h; calculated from data available in the papers, ** TOF [h^−1^].

## Data Availability

The datasets generated during and/or analysed during the current study are available in the RepOD repository https://doi.org/10.18150/MKTLDK.

## Supplementary Materials

The following supporting information can be downloaded at https://www.mdpi.com/article/10.3390/molecules30173600/s1, Figure S1: SEM image of the 7ZrOPr-S catalyst; Figure S2: XRD spectrum of the 7ZrOPr-S catalyst; Figure S3: TG curves of fresh catalyst and catalyst after reactions.
