# Supplementary material for: High Selectivity and Yield in Catalytic Transfer Hydrogenation of Furfural to Furfuryl Alcohol by Zirconium Propoxide Modified Mesoporous Silica
Source: Molecules. 2025 Sep 3;30(17):3600. doi: 10.3390/molecules30173600 (PMC12430864; doi:10.3390/molecules30173600)
Supplement: Supplementary file 1 [file molecules-30-03600-s001.zip › molecules-3829154-supplementary.pdf]

Supplementary Material

High selectivity and yield in catalytic transfer hydrogenation of furfural to furfuryl alcohol by zirconium propoxide modified mesoporous silica

Agnieszka Ciemięga[1]*, Katarzyna Maresz[1], Katarzyna Janoszka[2] and Julita Mrowiec-Białoń[1]

[1]Institute of Chemical Engineering of the Polish Academy of Sciences, Bałtycka 5, 44-100 Gliwice, Poland; ciemiega@iich.gliwice.pl, maresz@iich.gliwice.pl, j.bialon@iich.gliwice.pl

[2]Institute of Environmental Engineering of the Polish Academy of Sciences, M. Skłodowskiej-Curie 34, 41-819 Zabrze, Poland; katarzyna.janoszka@ipispan.edu.pl

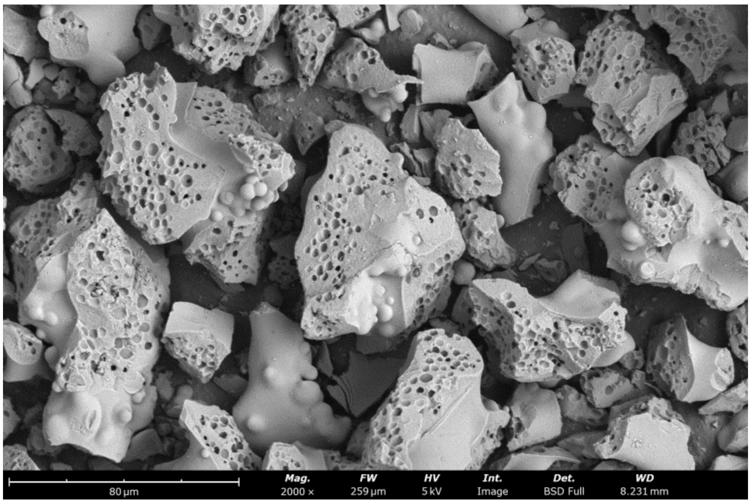

Figure S1. SEM image of the 7ZrOPr-S catalyst

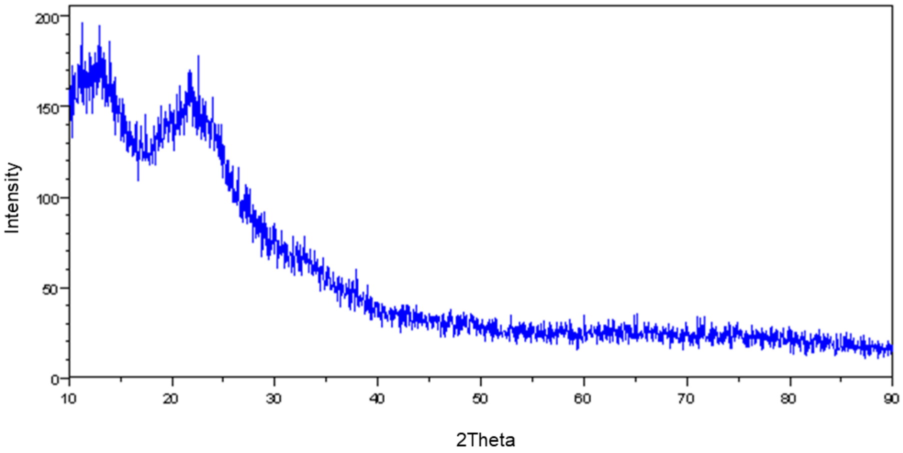

Figure S2. XRD spectrum of the 7ZrOPr-S catalyst

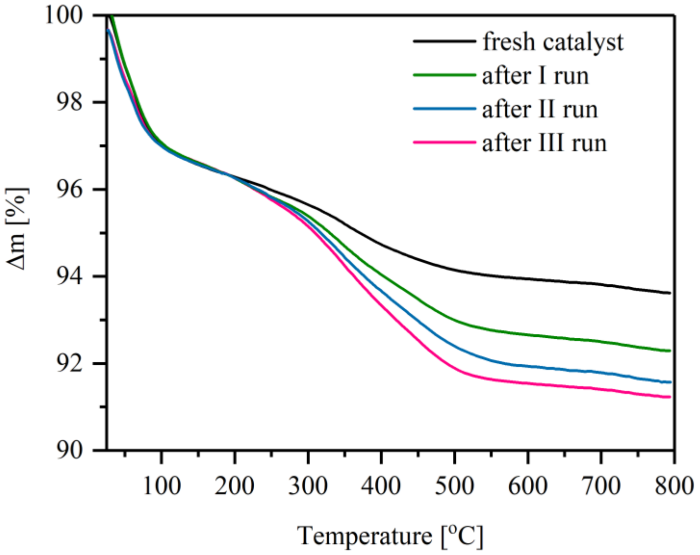

Figure S3. TG curves of fresh catalyst and catalyst after reactions
